# Fatal yellow fever among captive non-human primates in southern Colombia, 2025

**DOI:** 10.3389/fvets.2025.1655474

**Published:** 2025-08-21

**Authors:** Ivan Camilo Sanchez-Rojas, D. Katterine Bonilla-Aldana, Catherin Lorena Solarte-Jimenez, Jorge Luis Bonilla-Aldana, Marixa Belisario-Tovar, Sidaly Ortega-Gómez, Vilma Marielis Zambrano-Quenan, Julian Camilo Perafan-Gomez, Carlos Hernan Gomez-Ocampo, Mayerly Delgado-Cajigas, Alfonso J. Rodriguez-Morales

**Affiliations:** ^1^Grupo de Investigación en Recursos Naturales Amazónicos GRAM, Institución Universitaria del Putumayo, Mocoa, Colombia; ^2^College of Medicine, Korea University, Seoul, Republic of Korea; ^3^Grupo de Virologia, Universidad El Bosque, Bogotá, Colombia; ^4^Veterinarian Div., CEA CORPOAMAZONIA, Mocoa, Colombia; ^5^General Direction, Corporación para el Desarrollo Sostenible del Sur de la Amazonia (CORPOAMAZONIA), Mocoa, Colombia; ^6^Subdirection of Environmental Administration, Corporación para el Desarrollo Sostenible del Sur de la Amazonia (CORPOAMAZONIA), Mocoa, Colombia; ^7^Advisory Board Adjunct to General Direction, CORPOAMAZONIA, Mocoa, Colombia; ^8^Biology Div., CEA CORPOAMAZONIA, Mocoa, Colombia; ^9^Faculty of Health Sciences, Universidad Científica del Sur, Lima, Peru; ^10^Grupo de Investigación Biomedicina, Faculty of Medicine, Fundación Universitaria Autónoma de las Américas-Institución Universitaria Visión de las Américas, Pereira, Colombia

**Keywords:** *Ateles*, *Cebus*, *Lagothrix*, *Aotus*, flavivirus, yellow fever, non-human primates, Colombia

## Abstract

**Background:**

Yellow fever virus (YFV) remains a re-emerging zoonotic threat in South America. While epizootics in free-ranging *Alouatta* spp. are well-documented, little is known about YFV infection in other Neotropical non-human primates (NHPs), particularly in captive settings. Here, we report eight NHP fatalities associated with YFV occurring in early 2025, in the Colombian department of Putumayo, a known endemic area.

**Cases description:**

Between February and May 2025, eight fatal YFV cases were confirmed via RT-PCR in four NHP genera—*Cebus albifrons*, *Ateles fusciceps* (IUCN-endangered), *Lagothrix lagotricha* (vulnerable), and *Aotus* spp.—housed at wildlife centers or found nearby. Clinical signs included jaundice, lethargy, dyspnea, and mucosal pallor. Gross pathology revealed multisystemic involvement, with frequent hepatic necrosis, myocarditis, pulmonary edema, and severe parasitism. Histopathological examination in three representative cases identified hallmark features of yellow fever hepatitis: midzonal to centrilobular necrosis, Councilman bodies, steatosis, and sinusoidal congestion. These findings confirm fulminant YFV infection in previously undocumented captive primate hosts.

**Conclusion:**

This report presents the first evidence of natural YFV infection in *C. albifrons*, *A. fusciceps*, and *L. lagotricha* under managed care conditions. The presence of YFV in endangered and vulnerable NHPs has critical implications for conservation and public health. Epizootic surveillance protocols must expand beyond *Alouatta* spp. to include a broader range of species and captive populations. Reinforced vector control, biosafety measures, and One Health-based interventions are urgently needed to prevent spillover and enhance preparedness for future outbreaks.

## Introduction

South America is experiencing a worrying resurgence of yellow fever (YF) in 2024/2025 (1, 2). Up to August 14, 2025, 326 confirmed human cases have been reported across Bolivia, Brazil, Colombia, Ecuador, Guyana, and Peru, significantly evolving to severe disease, with a case fatality rate approaching 42% (136 deaths)^1^ (3, 4). Notably, the yellow fever virus (YFV) is spreading beyond traditional Amazonian zones into more populated areas such as São Paulo, Brazil, and Tolima, Colombia, increasing the risk of urban or periurban transmission (5–8). These outbreaks are being driven by sylvatic spillover from infected non-human primates (NHP) and persistently low vaccination coverage (9, 10), which remains below the threshold required for herd immunity (6, 11–14). In response, public health authorities are intensifying surveillance, laboratory testing, and both routine and emergency vaccination campaigns to contain the outbreaks and prevent further transmission across the region (15, 16).

Colombia is one of the most a ffected countries in South America due to YF (1, 2, 5, 8). Up to August 14, 2025, 130 cases have been confirmed (0.25 cases per 100,000 pop) (2.47 cases per 1,000,000 pop), with 55 deaths (42.31%)^2^ (17). Ten out of 32 departments (and the Capital District) are currently affected by YF during the 2024/2025 YFV outbreak. After Tolima (110 cases, with 40 of them, with fatal outcomes) (36%), Putumayo is the department with the highest number of confirmed cases, eight in total, with six fatal outcomes (75%). Four of those cases were reported early in the outbreak in 2024 (all of them fatal) (8). This department, located along the borders with Ecuador and Peru, far from Tolima (>500 kms), is historically significant for YFV and other vector-borne infections, posing ongoing risks due to cross-border mobility, dense rainforest ecosystems, and sylvatic vector presence (18–21). Putumayo is located in the south of the Eastern foothills of the central mountain range, which has been described as an area previously classified as endemic for sylvatic YF in Colombia (22).

In this context, epizootic surveillance is a critical component in the early detection and prevention of YF outbreaks in South America, where NHPs serve as critical sentinel species (6). Monitoring NHP morbidity and mortality enables the timely identification of viral circulation, guiding public health interventions such as targeted vaccination and vector control (23–26). Continued research on affected NHPs, including clinical, pathological, and molecular studies, is essential to deepen our understanding of virus ecology, transmission dynamics, and species susceptibility. Strengthening these efforts supports a One Health approach, protecting both human and wildlife populations from future outbreaks (27, 28).

So far, in South America, more than 168 epizootics have been investigated, especially in Brazil and Colombia^3^ (6). In Colombia, more than 55 of them have been reported, most of them affecting *Alouatta seniculus* (75.0%) in Tolima and Huila^4^ (6).

Despite the public health efforts to study epizootics and to better understand the impact of YFV infection in different contexts, there is a lack of published studies among captive NHPs in South America (29–31). Additionally, there is a lack of descriptions in the literature of YFV infection among *Cebus albifrons*, *Lagothrix lagotricha*, *and Ateles fusciceps* (an endangered species according to the International Union for Conservation of Nature, IUCN) NHP species. Then, here we described eight cases of YFV infection, confirmed by RT-PCR, among *Cebus albifrons, Lagothrix lagotricha, Ateles fusciceps*, and *Aotus* sp. NHPs were primarily found at our facilities, including the Corpoamazonia’s Wildlife Care and Assessment Center (*Centro de Atención y Valoración de Fauna*) (6 cases) (Supplementary Figure S1), and two were found at nearby locations.

## Cases description

Between February and May 2025, eight cases of fatal YFV infection were identified among captive and semi-captive NHPs in the department of Putumayo, southern Colombia. All cases were laboratory-confirmed via RT-PCR on tissue samples (Table 1). The affected individuals belonged to four different genera: *Cebus albifrons* (white-fronted capuchin), *Ateles fusciceps* (black-faced spider monkey), *Lagothrix lagotricha* (Humboldt’s wooly monkey), and *Aotus* spp. (night monkeys). Most individuals were housed in the Corpoamazonia’s Wildlife Care and Assessment Center (Centro de Atención y Valoración de Fauna) or the Suruma Amazonian Emblematic Fauna Park, with two *Aotus* spp. found deceased in the vicinity of Orito (Supplementary Figure S1).

**Table 1:** Main clinical, pathological, and molecular findings in NHP fatalities associated with yellow fever in Putumayo, Colombia, 2025.

#	Species and status in IUCN	Family	Age (years)	Sex	Date of death or euthanasia	Clinical signs	Necropsy findings	RT-PCR for YFV	Relevant notes
1	*Cebus albifrons* (White-fronted capuchin) (Least concern, IUCN)	Cebidae	Juvenile (3)	Male	May 6, 2025	Lethargy some hours before death, fever, anorexia, jaundice	Interstitial and alveolar edema, hyaline membranes, congestion, lung necrosis, severe parasitism (larvae and cysts of nematodes) in the intestines	Positive	Found dead in the facilities
2	*Ateles fusciceps* (Black-faced spider monkey) (Endangered species, IUCN)	Atelidae	Adult (10)	Female	May 18, 2025	Agonal, pale-icteric mucous membranes, high fever (39.8 °C)	Generalized jaundice, cerebral hemorrhages, meningitis, pulmonary edema, cardiomegaly, pericardial effusion, hepatic necrosis, mesenteric lymphadenopathy	Positive	Severe systemic involvement
3	*Ateles fusciceps* (Black-faced spider monkey) (Endangered species, IUCN)	Atelidae	Adult (12)	Female	May 6, 2025	Dyspnea, muscle weakness, dehydration, pale mucous membranes	Rough hair coat, biliary calculi, severe parasitism (larvae and cysts) in intestines, colonic gas, and fermentation	Positive	Euthanized due to an advanced clinical condition
4	*Ateles fusciceps* (Black-faced spider monkey) (Endangered species, IUCN)	Atelidae	Adult (12)	Female	May 6, 2025	Agonal, pale mucous membranes, vulvar discharge	Similar to #3, with biliary calculi, severe parasitism, gastritis, colonic gas, loss of muscle tone	Positive	Euthanized due to an advanced clinical condition
5	*Ateles fusciceps* (Black-faced spider monkey) (Endangered species, IUCN)	Atelidae	Adult	Female	May 31, 2025	Found dead, pale mucous membranes, mild jaundice, temperature 34.5 °C	Interstitial and intra-alveolar edema, generalized hemorrhage, cardiomegaly, icteric pericardial fluid, myocarditis, lingual ulcers, icteric mesentery, intestinal larvae, meningoencephalitis	Positive	Found dead in in the facilities
6	*Lagothrix lagotricha* (Humboldt’s wooly monkey) (Vulnerable species, IUCN)	Atelidae	Adult (12)	Male	May 11, 2025	Found dead, pale-icteric mucous membranes	Hepatic necrosis, icterus, gastric ulcers, pancreatic necrosis, severe parasitism in intestines (with larval perforations), intestinal necrosis, rough hair coat, cachexia	Positive	Poor body condition (2.5/5), severe icterus
7	*Aotus* sp. (Night monkeys, owl monkeys or douroucoulis) (from Endangered to Least-concern species, IUCN)	Aotidae	Adult	Male	May 31, 2025	Found dead, pale mucous membranes, mild jaundice, foamy nasal discharge with blood traces	Pulmonary edema, myocarditis, liver necrosis, meningoencephalitis, generalized hemorrhages	Positive	Found dead nearby, Orito, Putumayo
8	*Aotus* sp. (Night monkeys, owl monkeys or douroucoulis) (from Endangered to Least-concern species, IUCN)	Aotidae	Adult	Female	May 31, 2025	Found dead, pale mucous membranes, mild jaundice, foamy nasal discharge with blood traces	Pulmonary edema, myocarditis, liver necrosis, meningoencephalitis, generalized hemorrhages	Positive	Found dead nearby, Orito, Putumayo

NHP, no-human primates; RT-PCR for YFV, Reverse Transcriptase Polymerase Chain Reaction for Yellow Fever Virus from hepatic tissues (see Supplementary material for additional details); IUCN, International Union for Conservation of Nature. Cases 1–6 were found dead in the Corpoamazonia Wildlife Care and Assessment Center (Centro de Atención y Valoración de Fauna de Corpoamazonia, Putumayo). Cases 7 and 8, were found nearby, at Vereda El Naranjito (approximately 50 kms from Mocoa).

**Figure 1:**
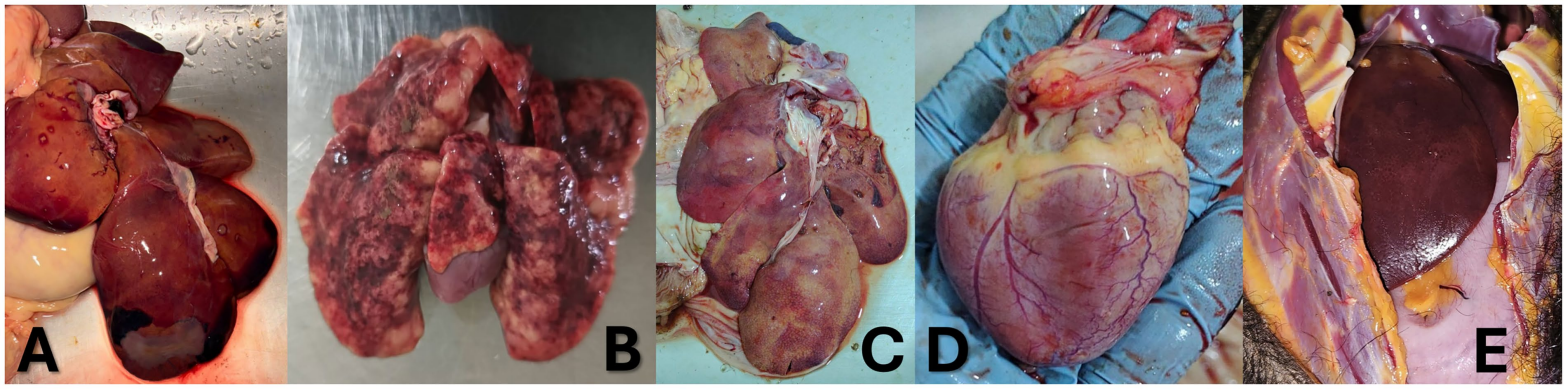
Appearance of organs of the dead NHPs with YFY infection. **(A)** Wooly monkey (*Lagothrix lagotricha*)—Liver. Hepatic necrosis is evident, predominantly affecting the centrilobular (zone 3) regions of the hepatic lobules. The tissue also exhibits discoloration consistent with jaundice. **(B)** White-faced capuchin monkey (*Cebus albifrons*), juvenile—Lung. There is prominent interstitial and intra-alveolar edema, with minimal epithelial damage. Occasional fibrinoid hyaline membranes are observed, along with generalized vascular congestion and scattered necrotic foci. **(C)** Black-headed spider monkey (*Ateles fusciceps*)—Liver. Marked discoloration and jaundice are present, accompanied by focal vascular congestion and architectural disruption, particularly in the right cranial hepatic lobe. **(D)** Black-headed spider monkey (*Ateles fusciceps*)—Heart. The myocardium shows yellowish discoloration (jaundice) with generalized vascular congestion, findings consistent with viral myocarditis. **(E)** Black-headed spider monkey (*Ateles fusciceps*)—Skin and subcutaneous tissue. Jaundice is evident in the subcutaneous fat and fascial connective tissue.

Case 1 involved a juvenile male *Cebus albifrons* presenting with lethargy, anorexia, jaundice, and fever before being found dead. Gross examination revealed pulmonary congestion, interstitial and alveolar edema, hyaline membrane formation, and necrotizing lung lesions. Severe intestinal parasitism, characterized by the presence of larvae and cysts, was also noted. Histological evaluation of the lung tissue (Figure 1B) confirmed edema and necrosis, with minimal epithelial damage, consistent with viral pneumonia (Table 1; Figure 1).

**Figure 2:**
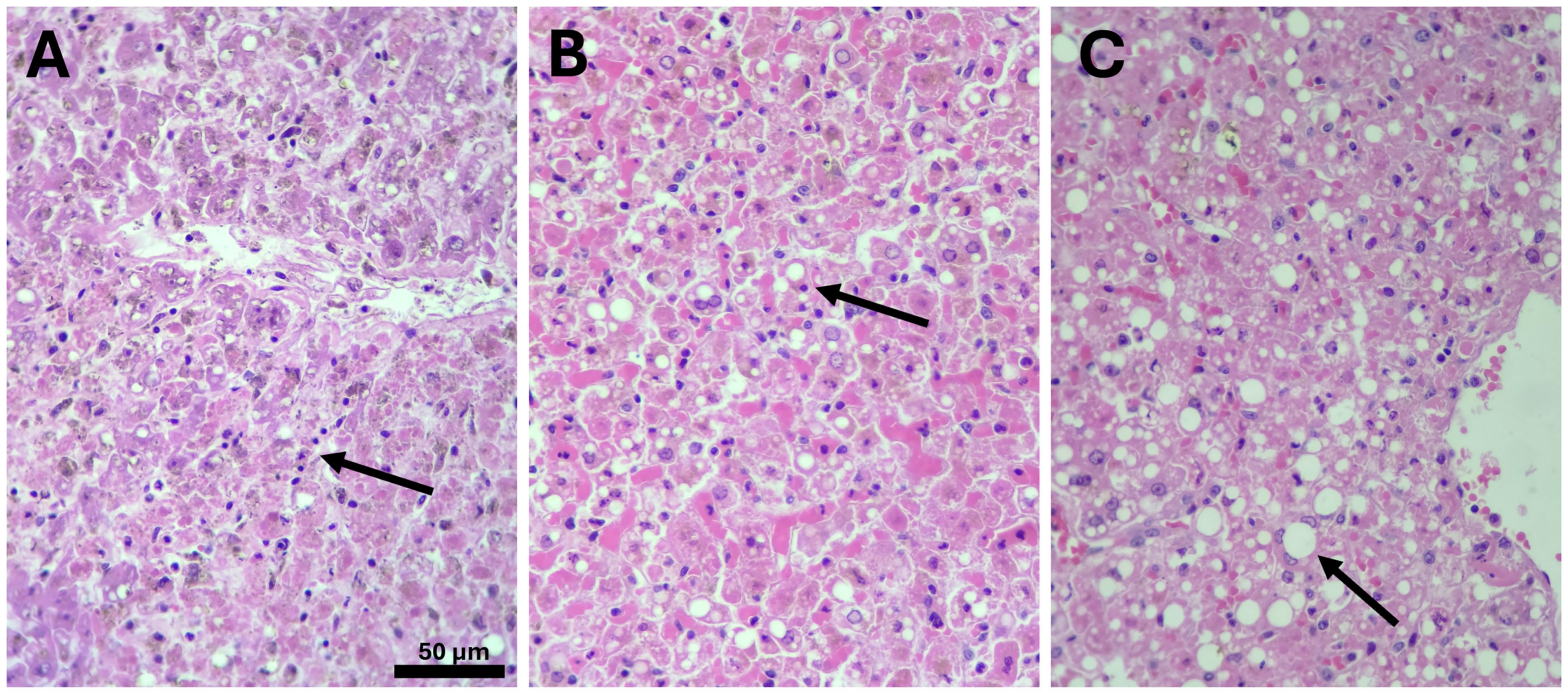
Histopathological findings at the liver of cases 5 (*Ateles fusciceps*) **(A)**, 7 **(B)**, and 8 **(C)** (*Aotus* sp.) [H&E-stained, 20X **(A)** and 40X **(B,C)**]. These histological images are strongly compatible with yellow fever virus-induced hepatitis, a condition that in non-human primates species typically presents with distinctive pathological features. Among these, midzonal (Zone 2) hepatocellular necrosis is a hallmark, often accompanied by numerous Councilman bodies, which represent apoptotic hepatocytes (arrows). Despite the extensive hepatocellular damage, the inflammatory response is characteristically minimal. Additionally, some specimens exhibit hepatocyte steatosis, which may reflect early injury or underlying metabolic stress associated with the YFV infection (Table 2).

Cases 2 to 5 involved adult female *Ateles fusciceps*, a species classified as endangered by the IUCN. They exhibited overlapping clinical signs such as mucosal pallor, jaundice, dehydration, vulvar discharge, and dyspnea. Body temperatures ranged from hypothermic (34.5 °C) to febrile (39.8 °C), and two monkeys were euthanized due to severe clinical deterioration. One of the monkeys had a poor nutritional status, and post-mortem scoring (see Supplementary material) indicated a low body condition (3/5). Common pathological findings included cardiomegaly, icteric mesentery, myocarditis, pulmonary edema, hepatic necrosis, gastrointestinal parasitism, and neurologic involvement (meningitis or meningoencephalitis). One individual (Case 5) had significant lingual ulceration and evidence of generalized hemorrhage (Table 1; Figures 1, 2).

Of these, Case 6 (*Lagothrix lagotricha*), an adult male, was found deceased with pale-icteric mucosa and marked cachexia. Necropsy revealed hepatic necrosis, gastric ulcers, pancreatic necrosis, and intestinal damage with evidence of larval perforation—an indication of concurrent severe parasitic burden. The animal had a poor nutritional status, and post-mortem scoring indicated a low body condition (2.5/5). Histologically, hepatic tissue showed centrilobular necrosis and marked discoloration consistent with jaundice (Table 1; Figure 1).

Cases 7 and 8, involving adult male and female *Aotus* spp., were discovered dead near the rural area of Vereda El Naranjito, Orito, near the Corpoamazonia facilities, in Mocoa (Supplementary Figure S1). Both presented with mild jaundice, foamy nasal discharge with blood traces, and generalized pallor. Both animals had a poor nutritional status, and post-mortem scoring indicated a low body condition (3/5). Pathological examination confirmed multi-organ involvement with prominent pulmonary edema, liver necrosis, myocarditis, and meningoencephalitis. These findings underscore the capacity of YFV to induce multisystemic and fulminant disease even in smaller-bodied nocturnal primates. Histopathological evaluation showed severe midzonal to centrilobular hepatocellular necrosis, Councilman bodies, steatosis, and sinusoidal congestion—hallmarks of YFV-induced hepatitis (Tables 1, 2; Figures 1, 2).

**Table 2:** Main histopathological findings in three of the cases.

Category	*Ateles fusciceps* (Case 5)	*Aotus* sp. (Case 7)	*Aotus* sp. (Case 8)
Zonal necrosis	Severe, midzonal and centrolobular	Severe, midzonal and centrolobular, extends focally to periportal	Severe, midzonal and centrolobular
Hepatocellular degeneration and necrosis	Ballooning degeneration, Councilman bodies (numerous), microvesicular vacuolation	Shrinkage and eosinophilic hepatocytes, Councilman bodies (moderate), micro- and macrovesicular steatosis	Micro- and macrovesicular steatosis, Councilman bodies (few), severe hepatocellular degeneration
Inflammatory infiltrate	Mild, lymphoplasmacytic and neutrophilic, multifocal (random and portal)	Mild to moderate, lymphoplasmacytic and histiocytic (random); occasional neutrophils	Mild, lymphoplasmacytic (periportal)
Sinusoidal and vascular changes	Severe multifocal congestion; leukocyte sequestration; mild portal vasculitis	Severe multifocal congestion; vascular wall infiltrate (mild); leukocyte sequestration	Moderate multifocal congestion; leukocyte sequestration
Kupffer cells/pigment	Activated Kupffer cells with brown granular pigment	Not specified	Brown granular pigment in hepatocytes and kupffer cells

Histopathological analysis, performed in three representative cases (Cases 5, 7, and 8), revealed consistent patterns of liver injury across species (Tables 1, 2; Figure 2). All showed midzonal necrosis with variable extension to centrilobular and periportal areas. The presence of Councilman bodies (apoptotic hepatocytes) ranged from a few to numerous. Hepatocellular steatosis, degeneration, and ballooning were also observed. Inflammatory infiltrates were mild and predominantly lymphoplasmacytic, with occasional histiocytes and neutrophils. Vascular changes included multifocal sinusoidal congestion and leukocyte sequestration, sometimes with mild vasculitis. Brown granular pigments, likely representing bile or iron, were detected in Kupffer cells and hepatocytes in two of the three animals (Table 2; Figure 2).

These findings align with classic YF pathology in primates and support the diagnosis of YFV infection as the primary cause of death. The involvement of multiple threatened and endangered species (*Ateles fusciceps*, *Lagothrix lagotricha*, and some *Aotus* spp.) emphasizes the ecological and conservation implications of the outbreak. Notably, most affected animals also had signs of severe parasitism, suggesting potential compounding effects of comorbidities on disease severity.

## Discussion

This cluster represents a rare and valuable documentation of YFV infection in captive NHPs under managed care in South America (32, 33). It highlights the vulnerability of non-*Alouatta* genera to fatal outcomes and underscores the importance of active epizootic surveillance, even in controlled environments (34, 35). Given the location of Putumayo along international borders and within endemic YFV transmission zones, these findings serve as a critical early warning for both public health and wildlife conservation authorities (8, 18–21). These threatened animals are often victims of illegal wildlife trafficking, a key issue that deserves emphasis and further documentation. Corpoamazonia, like others affected by the outbreak, has implemented prevention strategies and worked to conserve NHPs, fulfilling our duty to protect wild species.

Regarding *Cebus albifrons*, *Lagothrix lagotricha*, *Ateles fusciceps* (an endangered species according to the International Union for Conservation of Nature, IUCN), NHP species, we were not able to find in the biomedical literature reports of natural infection with YFV to any of these species. In the case of *Lagothrix lagotricha,* reports of experimental infection have been published before. In PubMed, only 50 publications in general about *L. lagotricha* can be currently retrieved. In 1930, a study found that *L. lagotricha* is relatively resistant to YFY (36). Only three individuals (25%) showed mild fever, none developed typical disease lesions, and virus transfer back to rhesus monkeys was rarely successful. However, their sera conferred protective immunity, indicating subclinical infection (36). No other study has been found regarding YFV and *L. lagotricha*. This species is susceptible to other infectious agents, including hepatitis B virus (36, 37), filarial nematodes (38), and *Toxoplasma gondii* (37).

In the case of *C. albifrons* and *A. fusciceps*, no reports at all of YFV infection (not even experimental) were found in the literature. In PubMed, only 110 publications in general about *C. albifrons* can be currently retrieved. This species is susceptible to other infectious agents, including *Leptospira* (38), *Trypanosoma* (39), and herpesvirus (40, 41), among others. In PubMed, only 38 publications in general about *A. fusciceps* can currently be retrieved. This species is susceptible to other infectious agents, including *Leptospira* (42), *Campylobacter hyointestinalis* (43), and SARS-CoV-2 (44), among others.

The detection of fatal YFV infection among captive individuals of *C. albifrons*, *L. lagotricha*, and *A. fusciceps* represents an unprecedented and alarming development. While *Alouatta* sp. have been extensively documented as highly susceptible to YFV and have historically served as sentinel species in South America, the involvement of other genera—especially those previously thought to be resistant or undocumented in natural conditions—calls for an urgent re-evaluation of current surveillance strategies (45–48). This outbreak reveals that reliance solely on *Alouatta* sp. as indicators of sylvatic YFV circulation may be insufficient in specific ecological settings or under evolving epidemiological pressures (6, 33, 49, 50).

The clinical and pathological findings in this report reaffirm the capacity of YFV to induce systemic, fulminant, and multisystemic disease, even in species with no known history of natural infection (50–52). The presence of characteristic histopathological lesions, including midzonal hepatic necrosis, Councilman bodies, myocarditis, and meningoencephalitis, supports the conclusion of YFV as the primary cause of death in these animals (53–55). Notably, several individuals showed concurrent evidence of severe parasitism and malnutrition, which may have contributed to increased susceptibility and worse clinical outcomes. These findings align with prior observations in other NHP epizootics in Brazil, where co-infections and stress-related immunosuppression potentially influenced mortality (14, 33, 56–58).

This outbreak must be interpreted in the context of the broader resurgence of YF in Colombia and South America (1, 2, 5–8, 11). In Colombia alone, more than 130 human cases and more than 50 deaths have been confirmed during the current outbreak. The department of Putumayo is among the most severely affected regions (8). Its location along the borders with Ecuador and Peru, coupled with dense rainforest ecosystems, high vector density, and frequent human-wildlife interactions, renders it a hotspot for arboviral emergence and re-emergence (18–21). In Brazil, according to the Ministry of Health, as of August 14, 2025, 87 cases of YFV infection have been confirmed in NHP in 70 municipalities. Notably, there is geographical overlap with human cases, particularly in the southern states affected by the disease.^5^

The role of captive NHPs in the epidemiology of YF must be carefully considered (6, 36, 48, 50, 53, 58). These animals are not only vulnerable to infection. Still, they may also serve as early indicators of viral circulation, particularly in areas where free-ranging NHPs are difficult to observe or are absent. Wildlife rescue centers, zoos, sanctuaries, and conservation parks thus represent valuable but underutilized platforms for syndromic and laboratory-based epizootic surveillance. The systematic incorporation of these facilities into national YF monitoring programs is strongly recommended (35, 59, 60).

Furthermore, this outbreak raises concerns about potential breaches in biosecurity and vector control within captive settings. Although it is unclear how the virus reached these managed populations, sylvatic vectors (*Haemagogus* spp., *Sabethes* spp.) likely entered the enclosures. In the case of this outbreak, the staff were not completely vaccinated before. Additionally, more than 30 tourists arrive daily at the center, and the vaccine certificate was not mandatory. However, such a possibility underscores the necessity for stringent mosquito control measures, enclosure design modifications (e.g., insect-proof netting), staff and visiting people vaccination, and regular entomological surveillance within and around such facilities (31, 61, 62).

In line with the OneHealth approach, this outbreak highlights the interconnectedness of human, animal, and environmental health. The death of NHPs in managed care settings is not only a tragic conservation loss—especially considering that *Ateles fusciceps* is an IUCN-listed endangered species—but also a sentinel signal for possible human spillover. Several historical YF outbreaks in South America have been preceded by or concurrent with NHP die-offs. Timely detection and reporting of these events are therefore critical for guiding human vaccination campaigns and vector control interventions (57, 63, 64). YF poses a serious threat to neotropical primates, notably howler monkeys, due to high mortality rates (65, 66).

Recommendations arising from this outbreak investigation include enhanced surveillance, with a focus on routine monitoring of all NHP species—not only *Alouatta* spp.—within epizootic surveillance protocols. This should encompass both free-ranging and captive populations, particularly in protected areas, wildlife rescue centers, and zoos (33, 63). Biosafety reinforcement in captive facilities is also essential; institutions housing NHPs must implement rigorous vector control strategies, such as physical barriers to prevent mosquito entry, environmental management to eliminate breeding sites, and indoor residual spraying where suitable. Vaccination of personnel working in close contact with NHPs in endemic regions should be prioritized, according to WHO guidelines, alongside regular health monitoring (10). Wildlife centers must have the capacity for rapid sample collection, preservation, and shipment to national reference laboratories, and the use of point-of-care diagnostics or field-deployable RT-PCR systems should be considered for timely detection (67, 68). Cross-sectoral collaboration between national public health and wildlife authorities is crucial for establishing integrated frameworks to detect, report, and respond to YFV epizootics. Finally, outbreak preparedness drills, including simulation exercises and staff training in captive facilities, should be conducted to ensure readiness for future epizootics, covering quarantine implementation and safe necropsy procedures (69, 70).

NHPs serve as vital sentinels for detecting YF outbreaks, and vaccination and eco-friendly vector control are key to prevention. The 17DD YF vaccine is safe and immunogenic in several neotropical primate species, though immune responses varied by species. Given the expanding geographic spread of YF in South America and its impact on NHP populations, it is crucial to intensify efforts aimed at protecting these species (65, 66).

From a scientific standpoint, this report recommends further research into the pathogenesis of YFV in understudied NHP genera. The susceptibility of *C. albifrons*, *A. fusciceps*, and *L. lagotricha*, in particular, deserves closer examination through experimental and immunological studies. The findings of subclinical or mild disease in early experimental infections, as reported in historical studies from 1930, contrast sharply with the fulminant presentations observed in this outbreak (36). Whether these differences are due to genetic factors, variations in viral strain, host immune status, or ecological pressures remains an open question (14, 63).

Moreover, the zoonotic risk of YF remains a pressing concern, particularly in the context of global warming, deforestation, and the expansion of human encroachment into wildlife habitats (6, 71–73). These drivers are likely to increase the frequency and reach of arboviral spillover events (74, 75). Thus, strengthening sentinel surveillance among NHPs—including those under human care—should be seen not as an isolated veterinary endeavor but as an essential component of public health strategy in the Amazonian and Andean regions (35).

We suggest including the environmental impact of YF on the ecosystem, emphasizing how viral persistence in NHPs may affect their evolutionary trajectory, an aspect crucial from our perspective as an environmental authority.

It is essential to stress the conservation implications of these findings. Beyond their role in disease ecology, NHPs are vital to the biodiversity and functioning of Neotropical forest ecosystems. Mass die-offs of species such as *A. fusciceps* may have long-term ecological consequences, including disruption of seed dispersal and forest regeneration dynamics. Their loss to YFV—potentially preventable through improved surveillance and protection—represents not only a failure in disease prevention but also in biodiversity conservation (76, 77). These findings may also have implications in other countries, such as Ecuador, Peru, Bolivia, and Brazil, as these species (*C. albifrons, A. fusciceps*, and *L. lagotricha*) (Supplementary Figure S1) are also present in these countries, where YF epidemics are currently occurring.

Finally, it is essential to emphasize that, while fatal YFV infections were observed in endangered and vulnerable NHP species, such as *Ateles fusciceps* and *Lagothrix lagotricha*, our findings do not confirm their role in sustaining YFV transmission cycles. Given the presence of comorbidities—including malnutrition and parasitism—it remains plausible that these cases reflect incidental infections rather than evidence of their participation as reservoir hosts. Caution must be exercised to avoid misinterpreting these events as indicative of a broader epidemiological role, particularly considering the conservation status of these species and the need to avoid undue alarm that may hinder their protection.

## Limitations

This investigation has several limitations. First, detailed histopathological studies could not be performed on all eight cases due to limitations in tissue availability and preservation, which restricted a more comprehensive pathological analysis across all species affected. Only three cases underwent histopathological examination, limiting the extrapolation of these findings to the broader group. Second, immunohistochemical analyses were not conducted, which would have provided more specific confirmation of YFV antigens within tissue lesions and further clarified the pathogenesis at the cellular level. Third, genomic studies, including the sequencing of viral strains, were not feasible due to resource and infrastructure constraints. As a result, we were unable to assess potential genetic variations in the circulating virus, which could influence virulence, host range, or transmission dynamics. These limitations underscore the need for strengthened diagnostic capabilities and comprehensive laboratory approaches in future outbreaks affecting NHPs in both wild and captive settings.

## Conclusion

In conclusion, this report presents novel and urgent data on the impact of the YF virus in previously undocumented NHP hosts in captivity. It underscores the need to broaden the taxonomic and ecological scope of YFV surveillance and to integrate One Health principles into regional outbreak response. The lessons learned from this cluster in Putumayo should inform national and international strategies to prevent, detect, and mitigate future YF outbreaks in both humans and NHPs.

## Data Availability

The original contributions presented in the study are included in the article/Supplementary material, further inquiries can be directed to the corresponding author.

## Footnotes

^1^ https://shiny.paho-phe.org/yellowfever/

^2^ https://www.ins.gov.co/Noticias/Paginas/fiebre-amarilla-datos-ciencia-prevencion.aspx

^3^ https://shiny.paho-phe.org/yellowfever/

^4^ https://www.who.int/news-room/events/detail/2025/06/11/default-calendar/who-epi-win-webinar-yellow-fever-in-the-americas-what-we-know

^5^ https://www.gov.br/saude/pt-br/composicao/svsa/cnie/painel-febre-amarela
