# Supplementary material for: Fatal yellow fever among captive non-human primates in southern Colombia, 2025
Source: Front Vet Sci. 2025 Aug 21;12:1655474. doi: 10.3389/fvets.2025.1655474 (PMC12409288; doi:10.3389/fvets.2025.1655474)
Supplement: Supplementary file 1 [file Supplementary_file_1.docx]

**Supplementary Material**

**Figure S1.** Geographical distribution of *Cebus albifrons*, *Ateles fusciceps*, and *Lagothrix lagothricha* in South America, focusing on the location of the Putumayo department and the Mocoa municipality in Colombia.


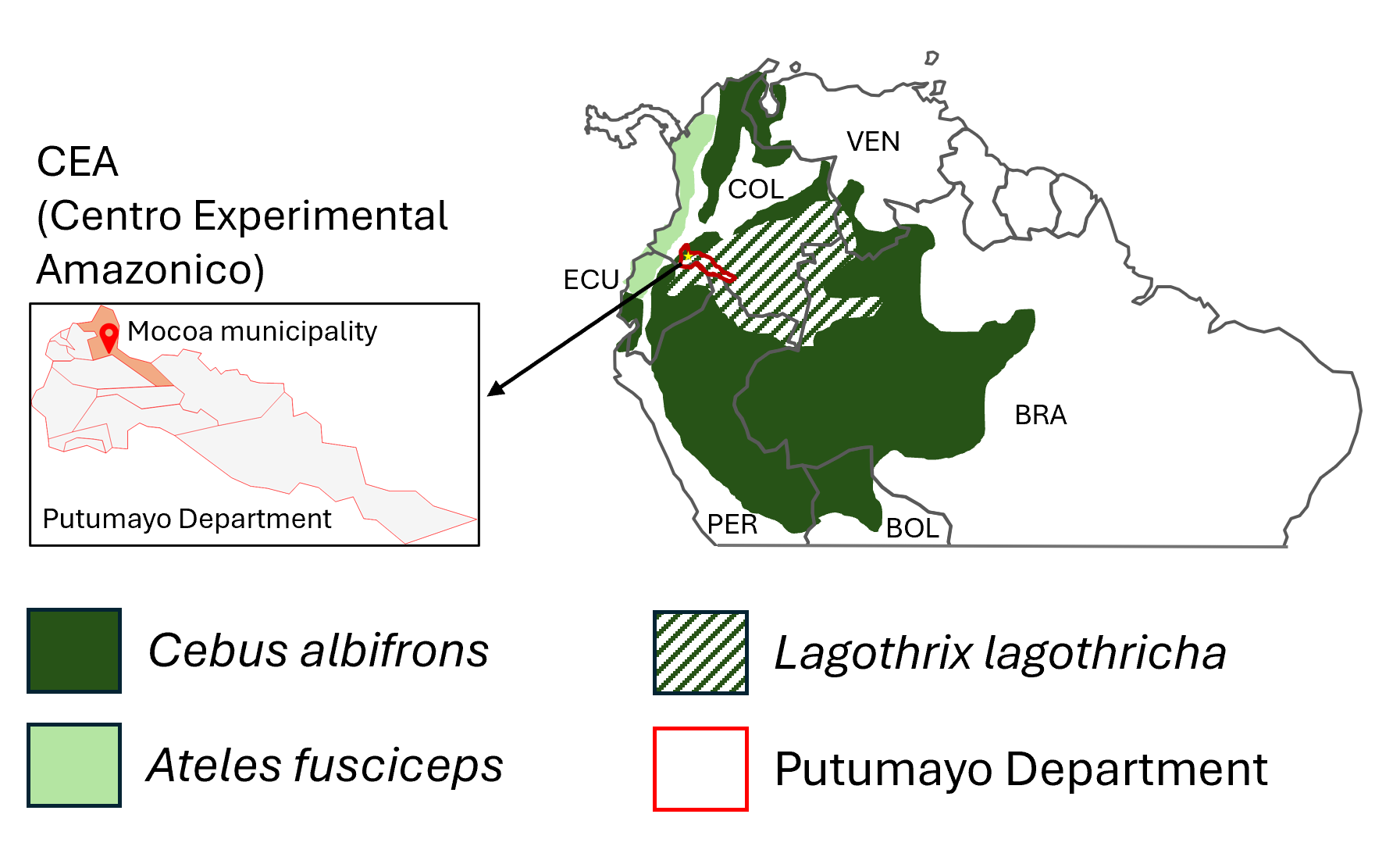


**Definition of post-mortem scoring for primate death**

Post-mortem scoring for primate death is a method to assess the stage of decomposition and estimate the time since death (PMI). A 1-5 scoring system, while not universally standardized, often reflects a scale from fresh (1) to extreme decomposition (5), with intermediate stages like early, advanced, and skeletonization.

Shrestha R, Kanchan T, Krishan K. Methods of Estimation of Time Since Death. [Updated 2023 May 30]. In: StatPearls [Internet]. Treasure Island (FL): StatPearls Publishing; 2025 Jan-. Available from: https://www.ncbi.nlm.nih.gov/books/NBK549867/

Franceschetti L, Amadasi A, Bugelli V, Bolsi G, Tsokos M. Estimation of Late Postmortem Interval: Where Do We Stand? A Literature Review. Biology (Basel). 2023 May 28;12(6):783. doi: 10.3390/biology12060783. PMID: 37372068; PMCID: PMC10295266.

**RT-PCR for Yellow Fever Virus**

Technique: Nucleic acid amplification.

Sample Temperature: -2.5°C

Method used: MEN-R01.5380-067 Detection of Arboviruses using real-time RT-PCR technique, for yellow fever virus.

Performed at the Laboratory of Virology of the National Institute of Health of Bogota, Colombia (national reference laboratory).
